# Prevalence of low back pain in adolescents with idiopathic scoliosis: a systematic review

**DOI:** 10.1186/s12998-017-0143-1

**Published:** 2017-04-20

**Authors:** Jean Théroux, Norman Stomski, Christopher J. Hodgetts, Ariane Ballard, Christelle Khadra, Sylvie Le May, Hubert Labelle

**Affiliations:** 1 Research Center, Sainte-Justine University Hospital Center, Montreal, QC Canada; 2 School of Health Profession, Murdoch University, 90, South Street, Murdoch, 6150 WA Australia; 3 Faculty of Nursing, University of Montreal, Montreal, QC Canada; 4 Faculty of Medicine, University of Montreal, Montreal, Canada

**Keywords:** Low back pain, Adolescent idiopathic scoliosis, Prevalence

## Abstract

**Background:**

Adolescent idiopathic scoliosis is the most common spinal deformity occurring in adolescents and its established prevalence varies from 2 to 3%. Adolescent idiopathic scoliosis has been identified as a potential risk factor for the development of low back pain in adolescents. The purpose of this study was to systematically review studies of the prevalence of low back pain in adolescents with idiopathic scoliosis in order to establish the quality of the evidence and determine whether the prevalence estimates could be statistically pooled.

**Methods:**

Systematic electronic searches were undertaken in PubMed, CINAHL, and CENTRAL without any restrictions. Studies were eligible for inclusion if they reported the prevalence of low back pain in adolescents with idiopathic scoliosis. Studies were excluded if they detailed the prevalence of pain in post-surgical subjects or were published in languages other than English or French. Data were reported qualitatively, since there was insufficient evidence for statistical pooling.

**Results:**

The electronic search strategies yielded 1811 unique studies. Only two studies fulfilled the eligibility criteria. The prevalence of low back pain in adolescents with idiopathic scoliosis ranged from 34.7 to 42.0%. However, these prevalence estimates should be viewed cautiously as the included studies were at high risk of bias.

**Conclusion:**

The results of this systematic review indicate that adolescents with idiopathic scoliosis frequently experience low back pain. However, there was insufficient evidence to confidently estimate low back pain prevalence in adolescents with idiopathic scoliosis and further studies are needed in this area.

**Electronic supplementary material:**

The online version of this article (doi:10.1186/s12998-017-0143-1) contains supplementary material, which is available to authorized users.

## Introduction

In developed countries, low back pain commonly occurs and is a leading cause of disability and financial burden [1]. The epidemiology of low back pain has been extensively researched in adults but is less well understood in adolescents [2]. Estimates of the prevalence of low back pain in adolescents vary widely, but a recent systematic review concluded that the 1-week prevalence was 17.7%, and 12-month prevalence was 33.6% [3]. Low back pain impacts significantly on adolescents, as over nine in ten report disability that may include reduced physical activity, school absenteeism, and limitations in daily activities [4–6].

The Scoliosis Research Society defines adolescent idiopathic scoliosis as a three-dimensional spinal deviation with a greater than 10° Cobb angle of unknown aetiology occurring in adolescents 10 years and older [7]. Adolescent idiopathic scoliosis is the most common spinal deformity occurring in adolescents and its established prevalence varies from 2 to 3% [8]. Adolescent idiopathic scoliosis has been identified as a potential risk factor for the development of low back pain in adolescents [9]. Subsequently, studies have been undertaken to establish the prevalence of low back pain in adolescents with idiopathic scoliosis.

Literature reviews have detailed the prevalence of back pain, but not specifically low back pain, in adolescents with idiopathic scoliosis [10]. Moreover, such reviews have not assessed the quality of the available evidence, and therefore have not established whether estimates of back pain prevalence may be subject to bias [10]. Hence, the purpose of this study was to systematically review studies examining the prevalence of low back pain in adolescents with idiopathic scoliosis in order to establish the quality of the evidence and determine whether the prevalence estimates could be statistically pooled.

## Methods

The methods used in this review accorded with PRISMA recommendations and the Methodological Evaluation of Observational Research guidelines [11, 12].

### Search strategy

**Fig. 1:**
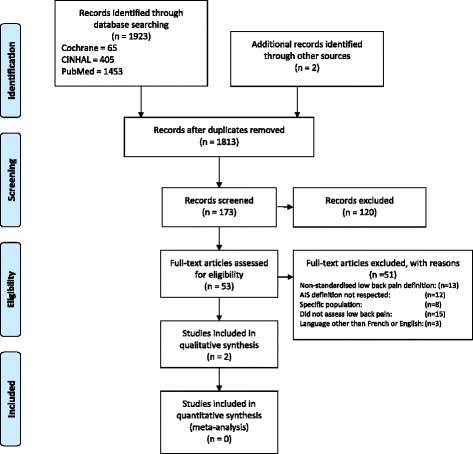
PRISMA diagram of the study selection process

Figure 1 displays the search strategies implementation and selection of studies. Database specific search strategies were developed to retrieve English or French language studies that detailed the prevalence of low back pain in adolescent idiopathic scoliosis populations. PubMed, CINAHL, and CENTRAL were searched without any restrictions. Additional file 1 presents the PubMed, CINAHL, and CENTRAL search strategies. The titles and abstracts of the studies retrieved through the searches were independently screened by two reviewers to identify potentially relevant studies that reported the prevalence of low back pain in adolescents with idiopathic scoliosis. In addition, citations in these identified studies were inspected to identify additional relevant studies. Two reviewers obtained full-text copies of all potentially relevant studies and considered them for inclusion in this review.

### Selection criteria

Any study that reported the prevalence of low back pain in adolescents with idiopathic scoliosis was considered for inclusion in this study. Adolescent idiopathic scoliosis was defined as a spinal deviation greater than 10° in adolescents aged between 10 and 17 years old [7]. Low back pain was defined as pain located on the posterior aspect of the body from the lower margin of the 12^th^ rib to the lower gluteal folds [13]. Studies were excluded if they detailed the prevalence of pain in post-surgical subjects or were published in languages other than English or French.

### Data extraction

One reviewer extracted data from the included studies into an Excel database. Only raw, aggregated prevalence data were extracted since the reported data in the included studies were not standardised or stratified by age, gender or other demographic characteristics. Low back pain case definitions were segmented according to minimum duration of low back pain episode and anatomic location of pain. Other extracted data included: study type, publication date, country, sample size, age, gender, prevalence period, Cobb angle, and data used to assess risk of bias.

### Assessment of risk of bias for included studies

Two reviewers assessed risk of bias for each included study, using a reliable purpose built tool [14]. This tool comprises 10 items that evaluate measurement bias, selection bias, and bias resulting from the analysis method. Discrepancies in the risk of bias assessment were resolved through consensus.

### Data analysis

All data were extracted into spreadsheets and then compared between reviewers to ensure consistency. Prevalence estimates were reported qualitatively since there was insufficient data for statistical pooling.

### Grading the strength of evidence

The GRADE Working Group grades of evidence were used to establish the overall strength of the evidence [15]. These grades are as follows:

- High quality: Further research very unlikely to alter confidence in the estimate of effect.
- Moderate quality: Further research likely to have an important impact on confidence in the estimate of effect and may change the estimate.
- Low quality: Further research very likely to have an important impact on confidence in the estimate of effect and is likely to change the estimate.
- Very low quality: Any estimate is very uncertain.

## Results

### Study selection

The electronic search strategies yielded 1811 unique studies (Fig. 1). Abstract and title screening identified 51 potentially relevant studies, for which full-text articles were retrieved and assessed for eligibility. An additional two potentially relevant studies were identified through inspection of the reference lists in the retrieved full-text articles. Of the retrieved full-text articles, two satisfied the eligibility criteria and were included in this review [16, 17].

### Study characteristics

Table 1 displays the included studies’ characteristics. All included studies used cross-sectional study designs and assessed low back pain through self-report questionnaires. In one study 85% of the respondents were female [16] and in the other study, all respondents were female [17]. The mean age of the respondents in one study was 14.2 years [16], and 14.7 years in the other study [17]. One study was conducted in Canada [16] and the other study was undertaken in Japan [17]. Neither of the prevalence estimates was standardised by age, gender, or other demographic characteristics.

**Table 1:** Characteristics of included studies

Country	Citation	Sample Size	Response Rate	Year of publication	Risk of bias	Study population	Case Definition	Prevalence period	Prevalence (%)	Standard error (%)
Canada	Theroux et al. [16]	500	Not Applicable	2016	High	Adolescents aged 10–17; 85% female	LBP >1 day	Point	42.0%	Not Reported
Japan	Makino et al. [17]	98	Not Applicable	2015	High	Adolescents; undefined age range; 100% female	LBP	1 week	34.7%	Not Reported

### Definitions of adolescent idiopathic scoliosis

Both included studies defined the eligibility criteria for adolescent idiopathic scoliosis as people aged 10–17 years with a Cobb angle of more than 10° [16, 17].

### Prevalence of low back pain and prevalence period

In one study the prevalence of low back pain was 42.0% with a prevalence period of the prior 24 h [16], and in the other study low back pain prevalence was 34.7% with a prevalence period of 1 week [17]. Neither of the included studies reported confidence intervals or other forms of error margins.

### Case definitions

Neither study reported minimum episode duration required to establish a case of low back pain. The anatomic location of pain was detailed as “low back pain” in one study [17] and “‘lumbar” in the other study [16], but neither study precisely specified the regions encompassed by these locations.

### Risk of bias

Table 2 summarises the risk of bias assessment. Both studies were at high risk of bias for five items.

**Table 2:** Risk of bias across the included studies

	Theroux et al. 2016 [16]	Makino et al. 2015 [17]
Study’s target population close representation of the national population for relevant variables.	High Risk	High Risk
Sampling frame a true or close representation of the target population	High Risk	High Risk
Random selection used to select the sample or census undertaken	High Risk	High Risk
Likelihood of non-response bias minimal	Low Risk	Low Risk
Data collected directly from the subjects	High Risk	High Risk
Acceptable case definition used in the study	High Risk	High Risk
Study instrument that measured the parameter of interest shown to have reliability and validity	Low Risk	Low Risk
Same mode of data collection used for all subjects	Low Risk	Low Risk
Length of the shortest prevalence period for the parameter of interest appropriate	Low Risk	Low Risk
Numerator and denominator for the parameter of interest appropriate	Low Risk	Low Risk

### Overall strength of the evidence

The overall strength of the evidence was considered to be very low quality, in terms of GRADE classifications.

## Discussion

This systematic review was the first to assess the prevalence of low back in adolescent idiopathic scoliosis. The findings show that the prevalence of low back pain in adolescents with idiopathic scoliosis ranges from 34.7 to 42.0%. However, the results of this review are based on a limited number of studies at substantial risk of bias. Subsequently, further studies are very likely to have a significant impact on estimates of low back pain prevalence in adolescents with idiopathic scoliosis.

Taken at face value, our findings suggest that over one-third of adolescents with idiopathic scoliosis experience low back pain. In contrast, a recent systematic review of low back pain in the general adolescent population found that the point prevalence was 12.0% and 1-week prevalence was 17.7% [3]. This tentatively suggests that adolescents with idiopathic scoliosis may be at least twice as likely to experience low back pain as adolescents without scoliosis. Again though, additional studies are required to improve the precision of estimates of low back pain in adolescents with idiopathic scoliosis.

The results of this systematic review have identified numerous methodological limitations in prevalence studies of pain in adolescents with idiopathic scoliosis. One such limitation involved the use of unclear definitions of low back pain. Some of the excluded studies reported the prevalence of back pain, but could not be included since it was unclear if the pain was located in the low back or other regions of the back. Hence, the location of back pain should be reported according to standardised anatomic definitions in subsequent studies [13].

Another limitation of low back pain prevalence studies in adolescent idiopathic scoliosis was the lack of longitudinal monitoring. Without such longitudinal studies, it cannot be established whether cases of low back pain are acute, brief and transitory, or if these cases are recurrent or chronic [18, 19]. It is important to establish whether cases of low back pain in adolescent scoliosis are chronic or recurrent since in these instances the impact on quality of life and burden are likely to be worse than in acute cases [20, 21]. Hence, longitudinal studies of low back pain in adolescent idiopathic scoliosis are warranted, and the findings should be reported by using standardised definitions of low back pain episodes [22, 23].

Notably omitted from the included studies was the presentation of confidence intervals or other forms of error margins. The lack of error margins makes it difficult to discern the precision of the low back pain prevalence estimates [24]. Also, the omission of error margins does not enable statistical pooling. Error margins can generally be estimated from other data presented in articles or data derived from other sources, but the interpretation of the prevalence estimates detailed in articles would be much enhanced by the presentation of error margins [24].

The lack of demographic factor segmentation was also a methodological shortcoming of the included studies. While one of the included studies had a gender distribution that was close to typical of the occurrence of idiopathic scoliosis in adolescents, the other study’s sample consisted completely of females. Such imbalances may bias the estimation of low back pain prevalence [25]. Also, none of the studies reported pain prevalence by age bands. If data were broken down by age segments, then it would be possible to establish the trajectory of low back pain prevalence in adolescent idiopathic scoliosis. Finally, low back pain prevalence in adolescent scoliosis should also be classified according to socio-economic status, as it has been associated low back pain in general adolescent populations [25, 26].

The final main limitation of the included studies that needs to be considered was the selection of study samples. In the included studies, the sample sizes were relatively modest and drawn from a single location. Such samples may lead to selection bias, and population-based studies are required to address this issue [25].

### Limitations

The findings of this review are likely to be influenced by publication bias that may have inflated the estimates of low back pain prevalence in adolescents with idiopathic scoliosis [27]. However, an extensive search was undertaken to identify potentially relevant studies, which should mitigate the impact of publication bias. Also, the risk of bias was assessed for each included prevalence estimate, and we have drawn careful, tentative conclusions on the basis of this assessment.

## Conclusion

The results of this systematic review indicate that adolescents with idiopathic scoliosis frequently experience low back pain. However, there was insufficient evidence to confidently estimate low back pain prevalence in adolescents with idiopathic scoliosis and further studies are needed in this area. Moreover, the studies included in this review had numerous methodological weaknesses that researchers should address in subsequent studies. Such limitations include the use of standardised definitions of anatomic low back pain location and episode durations; reporting of prevalence error margins; segmentation of low back pain prevalence by relevant demographic characteristics; monitoring of low back pain prevalence at regular intervals over extended periods; and the use of population-based study cohorts.

## Additional file

Additional file 1: Appendix 1. Search strategy. (DOCX 96 kb)
