# Supplementary material for: Prevalence of low back pain in adolescents with idiopathic scoliosis: a systematic review
Source: Chiropr Man Therap. 2017 Apr 20;25:10. doi: 10.1186/s12998-017-0143-1 (PMC5399433; doi:10.1186/s12998-017-0143-1)
Supplement: Additional file 1: — Appendix 1. Search strategy. (DOCX 96 kb) [file 12998_2017_143_MOESM1_ESM.docx]

Appendix 1

Search Strategy

**CINAHL Search Strategy**

TX thoracic pain OR TX lumbago OR TX dorsal pain OR TX mid-thoracic OR TX back pain OR TX chronic back pain OR TX acute back pain OR TX sacral pain OR TX sacroiliac pain OR TX thoracolumbar pain OR TX lumbar pain OR TX lumbopelvic pain OR (MH "Pain+") OR (MH "Back Pain+") OR (MH "Musculoskeletal Diseases+") AND TX rate OR TX incidence OR TX prevalence OR TX occurrence OR (MH "Epidemiology+") OR (MH "Prevalence") AND (MH "Adolescence+") OR (MH "Child+") OR TX juvenile OR (MH "Young Adult") AND (MH "Scoliosis+") OR (MH "Spinal Curvatures+") OR TX cobb angle

**PUBMED Search Strategy**

“thoracic pain” OR lumbago OR “dorsal pain” OR mid-thoracic OR “mid thoracic” OR “back pain” OR “chronic back pain” OR “acute back pain” OR “sacral pain” OR “sacroiliac pain” OR “thoracolumbar pain” OR “lumbar pain” OR “lumbopelvic pain” OR "Back Pain"[Mesh] OR "Musculoskeletal Diseases"[Mesh]) OR "Pain"[Mesh] AND "Epidemiology"[Mesh] OR "Prevalence"[Mesh] OR rate OR incidence OR prevalence OR occurrence AND "Adolescent"[Mesh] OR Adolescent* OR Adolescence OR Child* OR Juvenile OR "Young Adult"[Mesh] OR “Young Adult” OR “Young Person” OR “Young People” OR “Youth*” or “Teen*” AND Scoliosis[Mesh] OR “Cobb angle” OR "Spinal Curvatures"[Mesh] OR Scoliosis OR AIS

**Central Search Strategy**

#1 MeSH descriptor: [Back Pain] explode all trees

#2 MeSH descriptor: [Low Back Pain] explode all trees

#3 dorsal pain

#4 lumbago

#5 thoracic pain

#6 mid thoracic

#7 back pain

#8 chronic back pain

#9 MeSH descriptor: [Acute Pain] explode all trees

#10 acute back pain

#11 MeSH descriptor: [Myofascial Pain Syndromes] explode all trees

#12 sacral pain

#13 sacroiliac pain

#14 thoracolumbar pain

#15 MeSH descriptor: [Pain] explode all trees

#16 lumbopelvic pain

#17 MeSH descriptor: [Musculoskeletal Diseases] explode all trees

#18 #1 or #2 or #3 or #4 or #5 or #6 or #7 or #8 or #9 or #10 or #11 or #12 or #13 or #14 or #15 or #16 or #17

#19 MeSH descriptor: [Epidemiology] explode all trees

#20 Epidemiology

#21 MeSH descriptor: [Prevalence] explode all trees

#22 Prevalence

#23 MeSH descriptor: [Incidence] explode all trees

#24 incidence

#25 occurrence

#26 rate

#27 #19 or #20 or #21 or #22 or #23 or #24 or #25 or #26

#28 MeSH descriptor: [Adolescent] explode all trees

#29 Adolescent

#30 adolescence

#31 MeSH descriptor: [Young Adult] explode all trees

#32 Young Person

#33 Young People

#34 #28 or #29 or #30 or #31 or #32 or #33

#35 MeSH descriptor: [Scoliosis] explode all trees

#36 Cobb angle

#37 MeSH descriptor: [Spinal Curvatures] explode all trees

#38 AIS

#39 #35 or #36 or #37 or #38
